# The Epidemiology of Pediatric Autoimmune Hepatitis in Scotland: A National Cohort Study

**DOI:** 10.1097/PG9.0000000000000223

**Published:** 2022-07-29

**Authors:** Harry Sutton, Rachel Tayler, Iain Chalmers, Jennifer Cowieson, Karen Fraser, Paul Henderson, Richard Hansen

**Affiliations:** From the * Department of Paediatric Gastroenterology, Royal Hospital for Children, Glasgow, United Kingdom; † Department of Paediatric Gastroenterology and Nutrition, Royal Hospital for Children and Young People, Edinburgh, United Kingdom; ‡ Child Life and Health, University of Edinburgh, Edinburgh, United Kingdom; § Department of Paediatric Gastroenterology, Royal Aberdeen Children’s Hospital, Aberdeen, United Kingdom.

**Keywords:** autoimmune hepatitis, azathioprine, corticosteroids, liver transplant, prevalence

## Abstract

Autoimmune hepatitis (AIH) is a rare, but potentially severe, cause of liver disease in children. We aimed to summarize how children with AIH in Scotland presented, were investigated and managed in addition to producing novel epidemiological data and outcomes.

**Methods:**

All prevalent pediatric patients with AIH cared for in pediatric services between January 2013 and September 2018 were included. Individual patient data were obtained from electronic patient records in the 3-main academic pediatric centers in Scotland covering the entire population.

**Results:**

Thirty-eight patients were included (25 female) with median follow-up of 33 months (range, 2–145 mo) and 136 total patient years. The incidence between 2014 and 2017 was 0.49/100 000/y (95% confidence interval, 0.29-0.78) and point prevalence between 2013 and 2018 was 1.75/100 000 (95% confidence interval, 1.42-2.13). Thirty-five (92%) patients were autoantibody positive, most commonly anti-nuclear antibody (63%) and anti-smooth muscle antibody (42%). Thirty-seven (97%) patients had induction therapy with oral corticosteroids, 30 (79%) required maintenance treatment with azathioprine, and 23 (61%) received ursodeoxycholic acid. There were 1.4 disease flares per 10 patient years and 3 patients required liver transplantation with an overall 5-year survival rate without the need for transplantation of 95%.

**Conclusions:**

We calculated a novel incidence and prevalence rate for pediatric AIH in Scotland. Nearly all were invariably treated initially with corticosteroids with most placed-on azathioprine as maintenance therapy. Outcomes were generally favorable with low rates of disease flares and the need for transplantation being rare.

**What is Known**

- Autoimmune hepatitis (AIH) is a potential cause of liver cirrhosis and liver failure if left untreated.
- Previous studies have reported prevalence rates of 2.4–17.0 per 100 000 children.

**What Is New**

- The prevalence rate of AIH in children under 16 in Scotland is 1.75 per 100 000 children.
- The overall incidence of AIH was 0.49/100 000/y with type 1 AIH 0.44/100 000/y and type 2 AIH 0.05/100 000/y.
- Outcomes for children with AIH in Scotland were favorable overall with a 95% 5-year native liver survival.

## INTRODUCTION

Autoimmune hepatitis (AIH) is characterized by the presence of inflammation and fibrosis of the liver parenchyma and the intra- and/or extra-hepatic biliary tree associated with autoantibodies and raised IgG. It can lead to liver cirrhosis and potentially liver failure if left untreated (1). Its etiology is currently unknown but is believed to be multifactorial, with both environmental and genetic components interacting over time (2). AIH is typically managed with immunosuppression, although liver transplantation may be necessary when treatment fails or complications arise (3). AIH is the indication for liver transplantation in 2%–4% of pediatric cases, and the lifetime need for transplantation in AIH ranges between 9% and 55% (4). There is a close association between AIH and other autoimmune conditions (5), and the clinical presentation can be very variable (6). AIH is divided into 2 subtypes—AIH-1 and AIH-2, based on autoantibody positivity. Patients with AIH-1 show positivity for anti-nuclear antibody (ANA) and/or anti-smooth muscle antibody (SMA), while those with AIH-2 show positivity for anti-liver kidney microsomal type 1 antibody (anti-LKM-1) and/or anti-liver cytosol type 1 antibody (anti-LC-1) (6,7). Differences in presentation and outcomes between type 1 and type 2 have been reported (8).

There is currently a lack of research on the epidemiology and outcomes of pediatric AIH. Previous studies have reported prevalence rates from 2 to 17 per 100 000 children, with a female preponderance, and have reported incidence rates ranging from 0.23 to 0.60 per 100 000 children (9–11). In this study, we provide an overview of the presentation, initial investigation, management, and outcomes of all children with AIH in Scotland over a 5-year period. This allowed determination of population incidence and prevalence over this period with a captive denominator. We were also able to analyze the outcomes of these children with the ultimate goal of improving future management in the hopes of reducing disease flares and the need for liver transplantation.

## METHODS

### Setting

This was a multicenter, retrospective, audit of all AIH pediatric patients in Scotland over a 5-year period. Scotland is a devolved nation of the United Kingdom with a total population of 5.4 million people and 918 000 children under 16 years old (12). Scottish children with symptoms suggestive of AIH are referred from their local hospital or general practitioner to one of 3 tertiary pediatric gastroenterology centers in Scotland (Glasgow, Aberdeen, Edinburgh) for investigation, diagnosis and ongoing management. Additionally, children who move to Scotland after diagnosis in their home country have their care taken over by one of these 3 centers.

All 3 hospitals are operated through the National Health Service Scotland, the publicly funded single provider healthcare system for the country, which provides care free at the point of service. National Health Service Scotland is divided into smaller trusts, which are responsible for specific geographical areas.

### Patient Identification

All patients were diagnosed by their local centers as per local guidelines, with those diagnosed outside of Scotland meeting the relevant center’s diagnostic criteria (or being reevaluated if appropriate). Common criteria included biochemical, clinical, and/or histological evidence of hepatitis and the presence of SMA, ANA, and anti-LKM-1 positivity with elevated IgG level. Those patients born and diagnosed abroad were included for our general analysis and prevalence calculations but were excluded from incidence calculations.

Children with a diagnosis of AIH in Scotland between January 2013 and September 2018 were identified from prospectively populated incident databases at each site. A proforma for data collection was developed and the pediatric gastroenterology departments in Glasgow, Edinburgh, and Aberdeen provided the relevant patient data to encompass the whole population. Patients diagnosed after their 16th birthday were excluded. Data were obtained through retrospective review of electronic and paper records. The data were combined into a single dataset for analyses. The date of last follow-up was September 30, 2018, or the last clinical episode before transition/transfer/death. Follow-up duration was taken from the date of diagnosis regardless of whether the child was originally diagnosed in Scotland.

### Diagnostic Work-Up and Management

Individual patient work-up was not uniform across all centers, although variation was minimal and was based on the treating physician’s assessment. However, standard diagnostic investigations included baseline biochemistry, immunoglobulins, autoantibody profile, screening for other causes of chronic liver disease (eg, Wilson’s disease), and liver biopsy. Routine biochemistry and ultrasounds were repeated at regular intervals, at the discretion of the treating physician. Additional investigations included magnetic resonance cholangiopancreatographies (MRCPs), screening for inflammatory bowel disease (IBD) and celiac disease, and thiopurine methyltransferase (TPMT) genotyping and/or activity level. MRCP and liver biopsy specimens were reported by radiologists and pathologists from the patient’s local center, with a significant proportion of biopsies reviewed by one of the 3 UK quaternary Pediatric Hepatology centers.

The standard initial management of children with AIH was consistent across all 3 centers. Children were initially treated with 1–2 mg/kg (maximum dosing ranged from 40 to 60 mg) oral prednisolone, which was weaned over a course of 6–10 weeks depending on closely monitored biochemical response. Azathioprine was used as an additional maintenance therapy. At the time of management, patients were treated in accordance with the most up-to-date European Society of Paediatric Gastroenterology Hepatology and Nutrition guidelines (13). For this study, disease flares were diagnosed by a pediatric gastroenterologist and generally presented clinically as either newly developed or worsening symptoms/signs or rising transaminases. However, to allow better classification, we chose to categorize flares retrospectively by how they were managed: a mild flare was one that was managed with a nonmaximal steroid dose increase; a moderate flare managed with resuming maximal (2 mg/kg, maximum 60 mg) steroid dose; and a severe flare managed with in inpatient admission for intravenous (IV) methylprednisolone. Based on our review of the literature, there is currently no consensus on definition of disease flare; hence, our seeking to formally outline what we defined as a flare within the paper.

### At-Risk Population and Statistics

The unique Scotland-wide community health index number was used to identify patients and to avoid duplication. To allow for changes in population structure (although the total population of children <16 y in Scotland only rose by 0.87% from 2013 to 2018), incidence and prevalence rates were first calculated from official, publicly available, mid-year population figures, and then standardized to the 2018 mid-year estimate (12,14). Rates were then calculated using the direct method and 95% confidence intervals (CIs) were determined using a method based on gamma distribution (14). All rates are presented as per 100 000 of the at-risk population (ie, those < 16 y old). Point prevalence rates were calculated on July 1 annually from 2013 to 2018; incidence rates were calculated from 2014 to 2017 to ensure robust analysis. Patients were removed from any prevalence calculations once they reached their 16th birthday. All analyses were carried out using R (v3.1.1, R Foundation for Statistical Computing, Vienna, Austria) with the epitools package utilized where appropriate (15). A 2-tailed *P* value of less than 0.05 was considered significant in all analyses and 95% CIs given where appropriate. Biochemical values were expressed as percentages and medians ± range.

Institutional ethics approval was not sought for this research as all patient data was collected retrospectively as part of a national audit. All data were anonymized at the point of amalgamation.

## RESULTS

### Demographics and Epidemiology

Across Scotland, a total of 38 children were included (25 females; 66%). The median age at diagnosis was 11.4 years for all children (range, 1.0–15.9) and 11.4 and 12.0 for type 1 and 2, respectively. Children were followed up for a median of 33 months (range, 2–145 mo), totaling 136 patient years. Thirty-one (82%) were diagnosed as AIH type 1, while 7 (18%) had AIH type 2. Full antibody positivity is summarized in Table 1.

**TABLE 1.** Autoantibody positivity for children with type 1 and type 2 autoimmune hepatitis

Antibodies	Type 1, N = 31(82%)	Type 2, n = 7 (18%)
ANA	23 (74)	1 (14)
Anti-SMA	16 (52)	0
Anti-LKM-1	0	7 (100)
Anti-LC-1	1 (3)	1 (14)
Anti-MPO3	3 (10)	1 (14)
Anti-mitochondrial	0	1 (14)

ANA = anti-nuclear antibodies; LC = liver cytosol; LKM = liver kidney microsomal; MPO = myeloperoxidase; SMA = smooth muscle antibodies.

There were 2 patients with autoantibodies associated with both type 1 and type 2 AIH. The first patient was positive for SMA antibodies and anti-LC-1 antibodies. Although the most recent American Association for the Study of Liver Disease guidelines for diagnosis and management of AIH classifies anti-LC-1 antibodies as associated with type 2 AIH, it does not include it in its diagnostic requisites, stating “type 2 is characterized by the antibodies to anti-LKM-1, usually in the absence of ANA and SMA” (6). Because SMA positivity is part of the diagnostic requisite for type 1 AIH, the patient was classified as type 1. The second patient was positive for anti-LKM-1 but was also found to be weakly positive for ANA. As stated above, anti-LKM-1 is diagnostic of type 2 AIH, and although this is usually in the absence of ANA and SMA, this is not required. This patient was therefore classified as type 2.

The incidence of AIH between 2014 and 2017 was 0.49/100 000/y (95% CI, 0.29-0.78) with type 1 AIH 0.44/100 000/y (95% CI, 0.25-0.71) and type 2 AIH 0.05/100 000/y (95% CI, 0.01-0.20). Point prevalence between 2013 and 2018 was 1.75/100 000 (95% CI, 1.42-2.13).

### Presentation

Of the 38 children, 6 (16%) also had a member of their immediate family with an autoimmune condition. Eighteen (47%) had comorbidities, 14 of whom had IBD (37% of total). Eight (57%) of these 14 had ulcerative colitis, 5 (35%) had Crohn’s disease and 1 (7%) had colonic IBD type unclassified. Full demographics are summarized in Table 2. The most common clinical findings at diagnosis were jaundice, incidentally raised transaminases and abdominal pain, summarized in Table 3. The median duration of symptoms before presentation was 4 weeks (range, 0–72 wk).

**TABLE 2.** Demographic and follow-up patient data in all children and by autoimmune type

Variable	Type 1 (n = 31)	Type 2 (n = 7)	All (n = 38)
Age, y (range)	11.4 (1.5–15.9)	12.0 (1.0–15.2)	11.4 (1.0–15.9)
Female (%)	20 (64)	5 (71)	25 (66)
Follow-up time, mo (range)	32.5 (2–145)	62 (24–88)	33 (2–145)
IBD (%)	13 (42)	1 (14)	14 (36)
Positive AI family history* (%)	5 (16)	1 (14)	6 (16)
Comorbidities† (%)	17 (55)	1 (14)	18 (47)

AI = autoimmune; IBD = inflammatory bowel disease.

*AI family history included type 1 diabetes mellitus (n = 2), hyperthyroidism, hypothyroidism, multiple sclerosis, IBD, and celiac disease.

†Comorbidities included: celiac disease (n = 3), asthma (n = 2), sickle cell disease, idiopathic scoliosis, autoimmune hemolytic anemia, beta-thalassemia trait, and juvenile idiopathic arthritis.

**TABLE 3.** Presenting signs and symptoms for children with type 1 and type 2 autoimmune hepatitis

Presenting symptoms and signs	Type 1, N = 31 (%)	Type 2, n = 7 (%)
Jaundice	15 (48)	2 (28)
Incidental transaminitis	9 (29)	1 (14)
Abdominal pain	8 (25)	2 (28)
Fatigue	6 (20)	1 (14)
Colitic symptoms	8 (25)	0
Weight change	4 (13)	1 (14)
Vomiting	2 (6)	1 (14)
Dark urine	3 (10)	0
Organomegaly	0	1 (14)

In those who had IBD, 8 (57%) were diagnosed along with AIH during initial investigations, 3 (21%) were diagnosed prior (5 y, 2 mo, and 2 mo, respectively), and 3 were diagnosed after (6 mo, 1 y, and 10 y, respectively) to their AIH diagnosis. Five (36%) had overlap syndrome, 4 of whom had ulcerative colitis.

### Investigations

At initial presentation, all children had their transaminases, albumin, bilirubin, IgG, platelets, and antibodies checked. All children also underwent abdominal ultrasound and had their spleen size assessed. The median results at diagnosis and at 1-year post-diagnosis are summarized in Table 4.

**TABLE 4.** Median values with ranges of biochemical data and spleen size *z* score at diagnosis and 1-year follow-up

Investigation	At diagnosis, median (range)	At 1-y post-diagnosis, median (range)
Albumin (g/L)	35 (20–35) n = 35	41 (36–51) n = 33
ALT (IU/L)	444 (73–2429) n = 36	24 (11–73) n = 34
AST (IU/L)	376 (46–2308) n = 22	33 (17–66) n = 21
ALP (IU/L)	365 (158–896) n = 34	220 (72–501) n = 34
Bilirubin (umol/L)	92 (21–373) n = 37	NA
GGT (IU/L)	32 (1–194) n = 37	8 (3–20) n = 34
IgG (g/L)	22.25 (8.03–86.3) n = 36	13.7 (8.29–23.7) n = 19
Platelets (×10^9^/L)	240 (49–785) n = 37	234 (113–399) n = 34
Spleen size *z* score	3.67 (0.12–11.27) n = 24	1.85 (1.25–7.67) n = 19

ALP = alkaline phosphatase; ALT = alanine aminotransferase; AST = aspartate aminotransferase; GGT = gamma-glutamyltransferase; NA = not available.

Pathology reports were obtained from 35 (92%) of 38 children. Thirty-three (94%) of the biopsies were performed at one of the 3 tertiary Scottish centers, 2 biopsies were done abroad, and their reports were obtained by the Scottish institutions when care was taken over. Three children had no recorded biopsy, 2 of whom were not biopsied as they proceeded directly to transplant. Explant biopsy reports were not found during chart review. The third patient without an available liver biopsy report was diagnosed at an outside center, and so the biopsy report was not available on chart review but they tested strongly positive for ANA and anti-SMA. MRCP was included in the work-up of 31 (81%) children. MRCP identified findings consistent with overlap syndrome in 8 (21%) children, including stricturing and dilation of the biliary tract.

Additional work-up varied between patients. In 31 (82%) children anti-tissue transglutaminase (TTG) antibody was tested, 28 (73%) had TPMT genotyping and/or activity level (76% of those treated with azathioprine), 16 (42%) underwent endoscopic assessment, and 14 (37%) had a fecal calprotectin test. Two patients were found to be positive for anti-TTG antibody, 2 patients had signs of portal hypertension on baseline endoscopy and 8 patients had fecal calprotectin levels >200 μg/g, 7 of whom were diagnosed with IBD.

Of the 28 patients tested for TPMT, 26 (93%) had no variants detected or normal activity level. One patient had high TPMT activity and was not treated with azathioprine. The other had double point mutations at positions 460 and 719 (TPMT *3A genotype 1); they were treated with 75 mg of azathioprine. Their first 6-TGT level was 585.

### Outcomes

Oral steroids were used as therapy in 37 (97%) children, and 30 (79%) received azathioprine. One patient was switched from azathioprine to mycophenolate mofetil after a moderate disease flare and continued on mycophenolate mofetil without subsequent flares. Eight (21%) children required an inpatient admission for IV corticosteroids due to a severe disease flare post-diagnosis. The median length of IV treatment was 6 days (range, 4–10 d). In addition to immunosuppression, 23 (61%) patients were treated with ursodeoxycholic acid though this was eventually discontinued in 6.

Initial steroid dosing ranged from 1 to 4 mg/kg with maximum doses between 40 and 60 mg. Median maintenance dose of steroids was 5 mg (range, 2.5–10 mg) and was reached in a median of 13.5 weeks (range, 6–24 wk). All but 2 patients continued on low-dose maintenance steroids, both of whom were managed with azathioprine monotherapy.

In those with IBD and AIH, all but 3 were managed with combination low-dose steroids and azathioprine, the 3 others were managed with low-dose corticosteroid monotherapy (maintenance dosages ranged from 2.5 to 5 mg). One patient with Crohn’s disease was able to be weaned off steroids and managed with azathioprine alone.

Disease flares (clinical or biochemical deterioration) were managed by increasing the patient’s steroid dose. Four (10%) patients had mild disease flares, 7 (18%) had moderate flares, 6 (16%) had severe flares, and 20 (52%) patients had no flares during follow-up. There was an average of 1.4 flares per 10 patient years, and no patient had more than 2 disease flares. Three (7%) patients required liver transplantation. Two presented with acute-on-chronic liver failure and both were transplanted within 1 month of listing. One patient presented with decompensated liver failure 8 years after diagnosis and was transplanted after no improvement with antibiotics and IV steroids. Overall 5-year survival rate without the need for transplantation was 95%.

## DISCUSSION

In this retrospective, nationwide audit, the diagnosis, work-up, management, and disease course of all pediatric patients with AIH in Scotland over a 5-year period were reported. The majority of patients were diagnosed after several weeks of symptoms and had deranged blood investigations at the time of diagnosis. IBD was the predominant comorbidity in our cohort, affecting 37% of our patients; this was nearly double previously reported rates from Canada and London (10,16). The cause of this discrepancy is unclear but possibly reflects increased patient identification through initial work-up. Despite no standard guideline being in place, variation in patient work-up was minimal. Most patients responded well to management, with the majority having normalized liver function tests at 1-year post-diagnosis. The majority of patients had no disease flares, and no patient had more than 2 disease flares during follow-up. Only 3 patients required liver transplant, 2 of whom presented in acute-on-chronic liver failure.

We were able to calculate novel incidence and prevalence rates. To our knowledge, this is the first time a prevalence rate of AIH in children has been calculated in the United Kingdom. Our findings were similar to rates calculated in the United States (incidence 0.4, and prevalence 3.0 cases/100 000) and Canada (incidence 0.23, and prevalence 2.4 and 9.9 cases/100 000 for non-native and native children, respectively) (10,11,17).

The strength of our study comes from our population-based approach including all 3 pediatric gastroenterology centers in Scotland, effectively capturing all incident cases of AIH in the country between the years 2013 and 2018.

Establishing a standardized patient work-up and management can aid physicians dealing with rare diseases where they may have less experience. Our hope is to add to the available literature that can be called upon to create department protocols. Once a diagnosis of AIH has been established with positive antibodies and biopsy, there should be a baseline of additional investigations for these patients. Screening for the most common comorbidities such as celiac disease, with anti-TTG antibodies, autoimmune sclerosing cholangitis/overlap syndrome with MCRP and IBD with fecal calprotectin are all warranted, as these diagnoses may impact disease course and management of the patient. Screening for additional causes of liver dysfunction such as Wilson’s disease, drug-induced liver disease, viral hepatitis, and non-alcoholic fatty liver disease should also be included in the investigation work-up. Pretreatment genotyping and/or activity level of TPMT allows for the identification of patients at increased risk for developing severe myelosuppression if treated with a thiopurine (18). This should therefore be strongly considered during the initial work-up of these patients. A suggested complete set of investigations encompassing the diagnosis and these other considerations has been summarized in Supplemental Table 1 (http://links.lww.com/PG9/A87). This is now the standard of practice across Scotland.

Surveillance endoscopy for upper gastrointestinal varices is commonly done at many pediatric liver centers, although this remains controversial and is mainly reserved for those with established portal hypertension (19,20). Although not available at all of our centers, there is growing evidence for the use of elastography to monitor the development of fibrosis and cirrhosis (21,22). Current recommendations for its use in adult AIH are to wait 6 months from initiating treatment, as liver inflammation can impair its accuracy (23).

This study had several limitations. The retrospective design limited the scope of study. Additionally, although there was fairly consistent work-up and management of patients across the 3 centers, the multicenter approach meant we did not have uniformity in the choice of investigations undertaken and how disease flares were managed. Another significant limitation came in the MRCP and histology reports. Ideally, a single, radiologist/pathologist would review all MRCP images/biopsy samples in order to eliminate any inter-observer variability that may result from having multiple physicians reporting (24). The addition of formal fibrosis scoring across the cohort would also be useful but was too inconsistent to present here. Last, because the data were collected retrospectively not all results were available leading to missing data from some patients.

The diagnosis, work-up, and management of AIH in the adult population are well established but pediatric research on the topic remains limited. A study from a large pediatric liver center in the United States was recently published reporting the differences seen between their type 1 and type 2 AIH patients (8). Additional pediatric research is required across different settings and populations. Further reporting on pediatric AIH experience, particularly with regards to diagnosing, investigating, and managing the condition in children, would help unify management strategies with potential impact on clinical outcomes.

The development of a standard of care is a long-established practice. This can be difficult in the field of pediatrics, where there is a paucity of data. In 2018, the European Society of Paediatric Gastroenterology Hepatology and Nutrition Hepatology Committee published a position paper on the diagnosis and management of pediatric AI liver disease (13). These position papers, that help to guide management across the continent, are made possible by aggregating smaller studies, like the one we have presented. Here, we have reported the management and clinical course of all children diagnosed with AIH in Scotland, over a 5-year period. We have also reported novel incidence and prevalence rates. We hope this data can help guide the care of future patients with this rare and potentially severe disease.

## Supplementary Material



## References

[1] Christen U Hintermann E. Immunopathogenic mechanisms of autoimmune hepatitis: how much do we know from animal models? Int J Mol Sci. 2016;17:E2007. 10.3390/ijms17122007 PMC5187807 27916939

[2] Czaja AJ Manns MP. Advances in the diagnosis, pathogenesis, and management of autoimmune hepatitis. Gastroenterology. 2010;139:58–72.e4. 20451521 10.1053/j.gastro.2010.04.053

[3] Radhakrishnan KR Alkhouri N Worley S. Autoimmune hepatitis in children–impact of cirrhosis at presentation on natural history and long-term outcome. Dig Liver Dis. 2010;42:724–728. 20163994 10.1016/j.dld.2010.01.002

[4] Tanaka T Sugawara Y Kokudo N. Liver transplantation and autoimmune hepatitis. Intractable Rare Dis Res. 2015;4:33–38. 25674386 10.5582/irdr.2014.01034 PMC4322593

[5] Mieli-Vergani G Vergani D. Autoimmune paediatric liver disease. World J Gastroenterol. 2008;14:3360–3367. 18528933 10.3748/wjg.14.3360 PMC2716590

[6] Mack CL Adams D Assis DN. Diagnosis and management of autoimmune hepatitis in adults and children: 2019 practice guidance and guidelines from the American Association for the study of liver diseases. Hepatology. 2020;72:671–722. 31863477 10.1002/hep.31065

[7] Mieli-Vergani G Vergani D. Should giant cell hepatitis with autoimmune haemolythic anaemia be considered a paediatric autoimmune liver disease? J Pediatr Gastroenterol Nutr. 2018;66:e138. 29688999 10.1097/MPG.0000000000001911

[8] Roepe IG Vierling JM Goss JA. Presentation and outcomes of autoimmune hepatitis type 1 and type 2 in children: a single-center study. J Pediatr Gastroenterol Nutr. 2021;72:101–107. 32796427 10.1097/MPG.0000000000002892

[9] Verma S Torbenson M Thuluvath PJ. The impact of ethnicity on the natural history of autoimmune hepatitis. Hepatology. 2007;46:1828–1835. 17705297 10.1002/hep.21884

[10] Deneau M Jensen MK Holmen J. Primary sclerosing cholangitis, autoimmune hepatitis, and overlap in Utah children: epidemiology and natural history. Hepatology. 2013;58:1392–1400. 23686586 10.1002/hep.26454

[11] Jiménez-Rivera C Ling SC Ahmed N. Incidence and characteristics of autoimmune hepatitis. Pediatrics. 2015;136:e1237–e1248. 26482664 10.1542/peds.2015-0578

[12] Roughsedge E. Population Estimates Time Series Data. 2021. Available at: https://www.nrscotland.gov.uk/statistics-and-data/statistics/statistics-by-theme/population/population-estimates/mid-year-population-estimates/population-estimates-time-series-data. Accessed November 30, 2021.

[13] Mieli-Vergani G Vergani D Baumann U. Diagnosis and management of pediatric autoimmune liver disease: ESPGHAN hepatology committee position statement. J Pediatr Gastroenterol Nutr. 2018;66:345–360. 29356770 10.1097/MPG.0000000000001801

[14] Tripepi G Jager KJ Dekker FW. Stratification for confounding–part 2: direct and indirect standardization. Nephron Clin Pract. 2010;116:c322–c325. 20664287 10.1159/000319591

[15] Aragon TJ. epitools: Epidemiology Tools. 2021. Available at: https://cran.r-project.org/web/packages/epitools/index.html. Accessed November 30, 2021.

[16] Gregorio GV Portmann B Karani J. Autoimmune hepatitis/sclerosing cholangitis overlap syndrome in childhood: a 16-year prospective study. Hepatology. 2001;33:544–553. 11230733 10.1053/jhep.2001.22131

[17] Chung HV Riley M Ho JK. Retrospective review of pediatric and adult autoimmune hepatitis in two quaternary care centres in British Columbia: increased prevalence seen in British Columbia’s First Nations community. Can J Gastroenterol. 2007;21:565–568. 17853950 10.1155/2007/757906 PMC2657988

[18] Hindorf U Appell ML. Genotyping should be considered the primary choice for pre-treatment evaluation of thiopurine methyltransferase function. J Crohns Colitis. 2012;6:655–659. 22398041 10.1016/j.crohns.2011.11.014

[19] Sutton H Fitzpatrick E Davenport M. Transient elastography measurements of spleen stiffness as a predictor of clinically significant varices in children. J Pediatr Gastroenterol Nutr. 2018;67:446–451. 30234702 10.1097/MPG.0000000000002069

[20] Grammatikopoulos T McKiernan PJ Dhawan A. Portal hypertension and its management in children. Arch Dis Child. 2018;103:186–191. 28814423 10.1136/archdischild-2015-310022

[21] Mahmud N Doshi SD Forde KA. Transient elastography reliably estimates liver fibrosis in autoimmune hepatitis. Clin Exp Hepatol. 2019;5:244–249. 31598562 10.5114/ceh.2019.87639 PMC6781822

[22] Guo L Zheng L Hu L. Transient elastography (FibroScan) performs better than non-invasive markers in assessing liver fibrosis and cirrhosis in autoimmune hepatitis patients. Med Sci Monit. 2017;23:5106–5112. 29073121 10.12659/MSM.907300 PMC5669534

[23] Hartl J Denzer U Ehlken H. Transient elastography in autoimmune hepatitis: timing determines the impact of inflammation and fibrosis. J Hepatol. 2016;65:769–775. 27238753 10.1016/j.jhep.2016.05.023

[24] Kishanifarahani Z Ahadi M Kazeminejad B. Inter-observer variability in histomorphological evaluation of non-neoplastic liver biopsy tissue and impact of clinical information on final diagnosis in Shahid Beheshti University of medical sciences affiliated hospitals. Iran J Pathol. 2019;14:243–247. 31583002 10.30699/ijp.2019.99566.1985 PMC6742738

