# Supplementary material for: The Epidemiology of Pediatric Autoimmune Hepatitis in Scotland: A National Cohort Study
Source: JPGN Rep. 2022 Jul 29;3(3):e223. doi: 10.1097/PG9.0000000000000223 (PMC10158286; doi:10.1097/PG9.0000000000000223)
Supplement: Supplementary file 1 [file pg9-3-e223-s001.pdf]

**Diagnostic work-up**	Basic chemistry: liver function test (ALT, AST, ALP, Total and Direct Bilirubin, Albumin, GGT)<br>full blood count<br>urea and electrolytes<br>INR<br><br>Immunology: IgG level<br>ANA antibodies<br>SMA antibodies<br>Anti-LKM-1 antibodies<br>Anti-LC-1antibodies<br><br>Histology: liver biopsy<br><br>Imaging: Abdominal US with spleen size
**Additional Work-up**	Additional Imaging: Elastography (if available)<br><br>Screening: MRCP<br>Faecal calprotectin<br>Anti-TTG antibodies<br>Surveillance endoscopy (if indicated)<br><br>Screening for other liver diseases: DILI<br>Wilsons<br>Viral Hepatitis<br>A1AT deficiency<br>NAFLD<br><br>Management planning: TPMT genotyping and/or TPMT activity

Supplemental Table 1: Suggested diagnostic and additional work-up investigations for

autoimmune hepatitis in children.

AST: aspartate aminotransferase; ALT: alanine aminotransferase; ALP: alkaline aminotransferase; INR: International normalised ratio; IgG: immunoglobulin G; ANA: antinuclear antibodies; SMA: smooth muscle antibodies; LKM: liver kidney microsomal; LC: liver cytosol; US: ultrasound; MRCP: magnetic resonance cholangiopancreatography; TTG: tissue transglutaminase; DILI: drug induced liver injury; A1AT: alpha-1-antitrypsin; NAFLD: non-alcoholic fatty liver disease TPMT: thiopurine methyltransferase; GGT: gamma-glutamyltransferase
